# Neuropsychiatric aspects of long COVID: A comprehensive review

**DOI:** 10.1111/pcn.13508

**Published:** 2022-12-12

**Authors:** Takafumi Kubota, Naoto Kuroda, Daichi Sone

**Affiliations:** ^1^ Department of Neurology National Hospital Organization Sendai Medical Center Sendai Japan; ^2^ Department of Neurology Tohoku University Graduate School of Medicine Sendai Japan; ^3^ Department of Epileptology Tohoku University Graduate School of Medicine Sendai Japan; ^4^ Department of Pediatrics Wayne State University Detroit Michigan USA; ^5^ Department of Psychiatry Jikei University School of Medicine Tokyo Japan

**Keywords:** COVID‐19, long COVID, neurology, post COVID‐19 condition, psychiatry

## Abstract

Although some patients have persistent symptoms or develop new symptoms following coronavirus disease 2019 (COVID‐19) infection, neuropsychiatric aspects of long COVID are not well known. This review summarizes and provides an update on the neuropsychiatric dimensions of long COVID. Its neuropsychiatric manifestations commonly include fatigue, cognitive impairment, sleep disorders, depression, anxiety, and post‐traumatic stress disorder. There are no specific tests for long COVID, but some characteristic findings such as hypometabolism on positron emission tomography have been reported. The possible mechanisms of long COVID include inflammation, ischemic effects, direct viral invasion, and social and environmental changes. Some patient characteristics and the severity and complications of acute COVID‐19 infection may be associated with an increased risk of neuropsychiatric symptoms. Long COVID may resolve spontaneously or persist, depending on the type of neuropsychiatric symptoms. Although established treatments are lacking, various psychological and pharmacological treatments have been attempted. Vaccination against COVID‐19 infection plays a key role in the prevention of long coronavirus disease. With differences among the SARS‐CoV‐2 variants, including the omicron variant, the aspects of long COVID are likely to change in the future. Further studies clarifying the aspects of long COVID to develop effective treatments are warranted.

## Graphical abstract

Overview of neuropsychiatric aspects of long COVID.

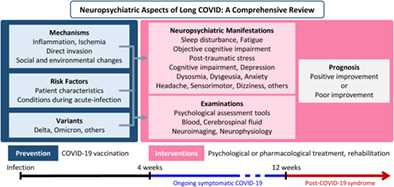

Coronavirus disease 2019 (COVID‐19) is a novel infectious disease and one of the most widespread infectious diseases in 2022.^1^, ^2^ COVID‐19 can severely damage multiple organs, including the nervous system, resulting in neuropsychiatric complications. The acute neurological complications of COVID‐19 include headache, anosmia, stroke, seizure, and encephalopathy.^3^

Although patients with COVID‐19 can recover completely, some have persistent symptoms or develop new symptoms following COVID‐19. The condition of persistent symptoms after COVID‐19 is termed ‘long COVID’ or ‘post‐COVID‐19 condition’.^4^, ^5^ Compared to the acute symptoms of COVID‐19,^3^, ^6^ the neuropsychiatric symptoms may be more common and severe in long COVID.^7^, ^8^ Many patients with long COVID suffer from fatigue, cognitive impairment, depression, anxiety, and sleep disorders.^7^, ^8^

However, the neuropsychiatric aspects of long COVID, including its clinical manifestations, mechanisms, examinations, risk factors, and interventions, are not well known. This review summarizes and provides an update on the neuropsychiatric dimensions of long COVID.

## Methods

This review is a narrative review. We conducted a search of MEDLINE (accessed from PubMed) up to 31 March 2022. In PubMed, the following key words (MeSH or in the title/abstract) were searched: (‘long COVID’[Title/Abstract] OR ‘post‐COVID‐19’[Title/Abstract] OR ‘post‐COVID’[Title/Abstract] OR ‘COVID long haulers’[Title/Abstract] OR ‘post‐acute COVID‐19 syndrome’[Title/Abstract]) AND (‘neurologic manifestations’[MeSH Terms] OR ‘neurological’[Title/Abstract] OR ‘brain’[Title/Abstract] OR ‘mental disorders’[MeSH Terms] OR ‘mental’[Title/Abstract] OR ‘psychiatric’[Title/Abstract] OR ‘psychological’[Title/Abstract] OR ‘headache’[MeSH Terms] OR ‘headache’[Title/Abstract] OR ‘cognition disorders’[MeSH Terms] OR ‘cognition’[Title/Abstract] OR ‘anosmia’[MeSH Terms] OR ‘anosmia’[Title/Abstract] OR ‘memory disorders’[MeSH Terms] OR ‘memory’[Title/Abstract] OR ‘anxiety’[MeSH Terms] OR ‘anxiety’[Title/Abstract] OR (‘depressive disorder’[MeSH Terms] OR ‘depression’[MeSH Terms]) OR ‘depression’[Title/Abstract] OR ‘sleep wake disorders’[MeSH Terms] OR ‘sleep’[Title/Abstract]). We screened the reference lists of all the relevant articles for additional data and conducted manual searching for relevant and latest research.

## Definition of Long COVID

Long COVID is a commonly used term to characterize signs and symptoms that persist or develop after acute COVID‐19, and the definition varies in each study. Long COVID may also be called post‐COVID condition, post‐acute sequelae of severe acute respiratory syndrome coronavirus 2 (SARS‐CoV‐2), post‐acute COVID‐19, or chronic COVID.^5^ However, the definition of long COVID by the National Institute for Health and Care Excellence includes ongoing symptomatic COVID‐19 and post‐COVID‐19 syndrome as follows^9^, ^10^, ^11^:

- Ongoing symptomatic COVID‐19: signs and symptoms of COVID‐19 from 4 to 12 weeks.
- Post‐COVID‐19 syndrome: signs and symptoms that develop during or after an infection consistent with COVID‐19, continue for more than 12 weeks and are not explained by an alternative diagnosis. It usually presents with clusters of symptoms, often overlapping, which can fluctuate and change over time and can affect any system in the body. Post‐COVID‐19 syndrome may be considered before 12 weeks while the possibility of an alternative underlying disease is also being assessed.

Therefore, this review includes both ongoing symptomatic COVID‐19 and post‐COVID‐19 syndrome.

## Neuropsychiatric Manifestations of Long COVID

Long COVID is common among patients with COVID‐19, and neuropsychiatric symptoms are among the most prevalent symptoms. In a systematic review and meta‐analysis (SRMA) focusing on persistent neuropsychiatric symptoms after COVID‐19 showed that the pooled prevalence of the neuropsychiatric symptoms was as follows: sleep disturbance, 27.4% (95% CI, 21.4%–34.4%); fatigue, 24.4% (95% CI, 17.5%–32.9%); objective cognitive impairment, 20.2% (95% CI, 10.3%–35.7%); anxiety, 19.1% (95% CI, 13.3%–26.8%); post‐traumatic stress disorder (PTSD), 15.7% (95% CI, 9.9%–24.1%); subjective cognitive impairment, 15.3% (95% CI, 8.9%–25.0%); depression, 12.9% (95% CI, 7.5%–21.5%); dysosmia, 11.4% (95% CI, 8.2%–15.6%); dysgeusia, 7.4% (95% CI, 4.7%–11.4%); headache, 6.6% (95% CI, 3.6%–12.0%); sensorimotor disturbance, 5.5% (95% CI, 2.4%–12.3%); and dizziness, 2.9% (95% CI, 1.6%–5.1%).^8^

The question may be raised whether long COVID‐like neuropsychiatric symptoms are specific to COVID‐19 patients. Many studies revealed the risk of neuropsychiatric symptoms is higher in COVID‐19 than in other infectious diseases.^12^, ^13^, ^14^, ^15^ In an analysis of 2‐year retrospective cohort studies including 1 284 437 patients, the risk of cognitive deficit and psychotic disorders remained higher after 2 years compared to patients with another respiratory infection.^12^ In a cohort study of 507 outpatients tested for COVID‐19, persistent symptoms were significantly more common in the COVID‐positive patients than in the COVID‐negative patients (223 [53%] vs 33 [37%]).^13^ Although these symptoms are also seen in other infections, their risk of occurrence is higher in COVID‐19 than in other infections. A retrospective cohort studies of 62 354 COVID‐19 cases without previous psychiatric disorders in the USA revealed that the incidence of the first psychiatric diagnosis in the following 14–90 days was higher than that in other infections such as influenza (hazard ratio [HR], 2.1; 95% CI, 1.8–2.5), other respiratory tract infections (HR, 1.7; 95% CI, 1.5–1.9), skin infections (HR, 1.6; 95% CI, 1.4–1.9), cholelithiasis (HR, 1.6; 95% CI, 1.3–1.9), and urolithiasis (HR, 2.2; 95% CI, 1.9–2.6).^14^ In a case–control study of 355 post‐COVID patients and 272 post‐sepsis patients, fatigue and depression were significantly more common in the former than in the latter, but there were no significant differences in cognitive impairment.^15^ These findings suggest that COVID‐19 has significant long‐term effects on the nervous system.

With constantly changing COVID‐19 variants, it is important to note the difference in long COVID symptoms between them. Since the long COVID risk with the omicron variant is half of that with the delta variant,^16^ incidence of long COVID may be less frequent with the former. On the other hand, another study found that the risk of neuropsychiatric symptoms in long COVID was similar for the delta and omicron variants.^12^ Considering the differences among variants, the presentation of long COVID is likely to change in the future; therefore, the changes in these variants should be closely monitored.

### Fatigue

Fatigue was the most common symptoms in patients with long COVID, with a prevalence of 24.4%–58%.^7^, ^8^ As an example of fatigue in a patient with long COVID, a previous active patient with long COVID were not able to go for long walks, climb hills, travel long distance, and lost her appetite due to fatigue.^17^ Since there are differences in the reported prevalence between studies, we focused on the most relevant studies. In a prospective cohort study in the USA, 55% (104/183) of the patients reported fatigue 35 days after hospitalization for COVID‐19. Moreover, fatigue of long COVID has serious impact on patient's life. In an ambidirectional cohort study of 2469 patients conducted 6 months after discharge in China, 63% patients had fatigue or muscle pain along with a negative impact on their work.^18^ During a 7‐month follow‐up study of COVID‐19 in the UK, 45.2% of the patients showed reduced work time compared to that before the disease, and 22.3% were not working due to health issues.^19^

Some studies investigated the prognosis of fatigue related to long COVID and revealed that fatigue associated with long COVID can continue for over 1 year.^20^, ^21^, ^22^ In a cross‐sectional study of 156 patients in the USA followed up for approximately 1 year after COVID‐19, 82% of them had fatigue, and the performance of physical activities of moderate and vigorous intensities was lower than that before COVID‐19. In addition, long COVID symptoms, including fatigue, were exacerbated by physical exertion, dehydration, whether changes, consuming large meals, premenstrual period, and alcohol consumption.^20^ Similarly, in a cohort study of 303 inpatients and outpatients in Italy after 1 year since the disease onset, fatigue was persistent in 52% of the patients.^22^ In contrast, in a prospective longitudinal study conducted 1 year after hospitalization for COVID‐19 in the USA, only 10% of the patients experienced fatigue.^21^

### Sleep disturbance

Sleep disturbance was one of most common neuropsychiatric symptoms in patients with long COVID, with a prevalence of 27.4% (95% CI, 21.4%–34.4%).^8^ As a patient experience of insomnia due to long COVID‐19, he was not able to get a full night's sleep where he felt like he was rested.^23^ Furthermore, a multicenter study of 1142 patients with COVID‐19 7 months after discharge in Spain showed that 34.5% of them had poor sleep quality (Pittsburgh Sleep Quality Index score ≥ 8).^24^ In a cohort study of 172 consecutive COVID‐19 survivors admitted to the intensive care unit (ICU) with acute respiratory distress syndrome, 60.5% of the patients had poor sleep quality 3 months after discharge, which was further confirmed by actigraphy.^25^ Sleep disturbance in long COVID may be caused by immunological effects, preexisting conditions, severe COVID‐19 infection,^26^ but persistent cough^7^ as a risk factor of insomnia^27^, ^28^ might also be associated with sleep problems.

Although it is not known how long the sleep disturbance persists after COVID‐19, another study showed that 10% of the patients hospitalized for COVID‐19 had poor sleep quality even 12 months after discharge.^21^ In addition to insomnia, the association between long COVID and circadian rhythm disorders has also been clarified. In Ukraine, a retrospective cohort study of 278 patients conducted 7.6 ± 1.1 weeks after COVID‐19 revealed that delayed sleep phase disorders were associated with COVID‐19 but not advanced sleep phase disorders, irregular sleep phase disorders, and non‐24‐h circadian rhythm disorders.^29^

### Cognitive impairment

Cognitive impairment symptoms in long COVID, such as brain fog, attention loss, and memory impairment, are common and negatively affect the patient's life.^30^ Although brain fog is a not medical word, it is frequently used to describe characteristic symptoms such as poor concentration, fuzziness of thought, confusion, slowed thinking, and mental fatigue.^31^, ^32^ In an SRMA focusing on persistent neuropsychiatric symptoms after COVID‐19, the prevalence of subjective and objective cognitive impairment was 15.3% (95% CI, 8.9%–25.0%) and 20.2% (95% CI, 10.3%–35.7%), respectively.^8^

Some studies have clarified the profile of cognitive impairment. Executive function was commonly impaired and other domains such as memory, attention, and learning were also disturbed. In a cross‐sectional study of 72 mild‐to‐moderate COVID‐19 survivors at average 4 months after the infection in the USA, 40% cases had cognitive impairment especially in the area of executive functioning.^33^ A case–control study of 2‐ and 10‐month follow‐up periods after hospital discharge in Italy investigated the change in cognition. At the 2‐month follow‐up, 53% of the patients had cognitive impairment, with a main deficit in the executive functions. Although 25% showed multi‐domain dysfunction, 16%, 6%, and 6% of the cases had a pure executive, memory, and visual–spatial impairments, respectively. At the 10 months follow‐up, 36% still had cognitive impairment, with 21% being multi‐domain, 6% being memory, 6% being visual–spatial, and 3% being pure executive.^34^ In a cohort study of 29 COVID‐19 patients conducted 4 months after hospital discharge in Denmark, approximately 60% of the patients had cognitive dysfunction, with verbal learning and executive functions being the most impaired. Moreover, objective cognitive impairment correlated with subjective cognitive complaints, poor work function, and reduced quality of life.^35^ In a cross‐sectional study of 18 young patients in Germany with a median recovery period of 85 days from mild to moderate COVID‐19, short‐term memory, attention, and concentration were particularly affected by COVID‐19.^36^ In a cohort study of 63 patients having subjective cognitive complaints at average 187 days after COVID‐19 in Spain, attention was most impaired, followed by executive functions. Interestingly, attention was not only affected alone but also in conjunction with impaired performance in executive functions, learning, and long‐term memory.^37^

Some longitudinal studies investigated the duration of cognitive dysfunction and found that it may not be permanent. Among the control, acute COVID (<1 month), post‐acute sequence of COVID‐19 (PASC) (1–4 months), and post‐PASC (>4 months) groups, selective impairment of attention was noted in the PASC group, with significant disturbance in the executive functions but normal alerting and orienting abilities.^38^ In a study of 78 COVID‐19 patients in Ecuador, although the score of Montreal Cognitive Assessment (MoCA) at the 6‐month follow‐up was significantly lower in patients with COVID‐19 than in those without, there was no difference at the 18‐month follow‐up.^39^ In a case–control study, there was no significant cognitive impairment reported after 6–9 months, suggesting evidence of recovery over time.^40^ Likewise, a prospective longitudinal study of COVID‐19 in the USA after 1 year of hospitalization showed significant improvements in the telephonic MoCA score (56% improvement, median 1 point, *P* = 0.002) and Neuro‐Quality of Life anxiety score (45% improvement, *P* = 0.003) from 6 to 12 months.^21^

### Depression and anxiety

Depression and anxiety are also serious issues in patients with long COVID and were seen at 12.9% (95% CI, 7.5%–21.5%) and 19.1% (95% CI, 13.3%–26.8%), respectively.^8^ In an SR focusing on depression in post‐COVID‐19 syndrome, 3%–12% of the cases presented severe depressive symptoms and/or clinically significant depression based on the Diagnostic and Statistical Manual of Mental Disorders (DSM)‐5 criteria, Beck Depression Inventory‐13 score ≥ 9, Patient Health Questionnaire (PHQ)‐9 score > 14, or Hospital Anxiety and Depression Scale‐Depression (HADS‐D) score > 10.^41^ For instance, a multicenter study of 1142 patients after a mean period of 7 months since hospitalization in Spain showed that the prevalence of depression and anxiety was 19.7% (HADS‐D ≥ 10 points) and 16.2% (HADS‐anxiety ≥ 12 points), respectively.^24^

Some studies investigated the changes and prognosis in depression/anxiety associated with long COVID with unfavorable results.^21^, ^42^, ^43^ In the longitudinal observational study of 239 patients in the Netherlands, there was no significant improvement in the depressive symptoms at the 3‐ and 6‐month follow‐up.^42^ A multicenter cohort study in Spain revealed that the incidence of anxiety symptoms 12 months after hospitalization was significantly higher in the COVID‐19 patients than in the matched patients hospitalized for other causes.^43^ Similarly, a prospective longitudinal study conducted in the USA on COVID‐19 patients after 1 year of hospitalization reported no significant improvement in high anxiety and depression between 6 and 12 months. Moreover, the prevalence of high anxiety and depression at 12 months was 7% and 4%, respectively.^21^ Hence, health care workers and families should closely monitor patient's depressive and anxious feeling and keep supporting them.

### PTSD

As PTSD can develop in patients following severe infections such as Severe Acute Respiratory Syndrome and Middle East Respiratory Syndrome, patients with long COVID also experience PTSD.^44^ Approximately 10.5%–37.2% of long COVID patients developed PTSD.^42^, ^45^, ^46^, ^47^, ^48^, ^49^ As symptoms of PTSD, patients after COVID‐19 infection may have intrusive thoughts or images of COVID‐19 related traumatic event, avoidance of whatever reminds them of the event, or changes in the way emotions are experienced.^50^ PTSD after COVID‐19 were seen in health care workers.^51^

Several studies have reported prognosis in the PTSD symptoms during the follow‐up period with opposite results.^42^, ^47^ In a longitudinal observational study of 239 patients in the Netherlands, the prevalence of PTSD (Trauma Screening Questionnaire [TSQ] ≥ 6 points) significantly improved from 37.2% at 3 months to 26.8% at 6 months since the onset of COVID‐19 symptoms. Of the TSQ items, difficulty in concentrating, difficulty falling or staying asleep, and upsetting thoughts/memories were common symptoms at 3 months after the onset of COVID‐19. Between 3 and 6 months, heightened awareness of potential dangers, difficulty falling or staying asleep, bodily reactions, and upsetting thoughts/memories significantly improved. In contrast, in an online survey of 3290 COVID‐19 patients in the UK, the prevalence of PTSD worsened gradually and significantly with 15% patients reporting symptoms at 4–8 weeks, 17.3% at 8–12 weeks, and 18.9% at >12 weeks.^47^

### Impact on work and life

Long COVID makes a serious impact on patients' work and life. In an international cohort study, around 20% of patients were not working and nearly half of patients reduced their working time due to a direct effect of long COVID.^19^ In particular, patients with cognitive dysfunction associated with long COVID likely have serious effects on their life and work, with 88% having difficulty working, around 75% having difficulty making serious decisions, around 55% having difficulty driving, and around 55% having difficulty following simple instructions.^19^ Similarly, in a case–control study of Denmark, objective cognitive impairment correlated with poor work function and reduced quality of life.^35^

## Examinations of Long COVID

### Psychological assessment

As most neuropsychiatric symptoms of long COVID are subjective, appropriate measurement using psychological scales is crucial. Generally, relatively common scales for several cognitive domains have been utilized to assess the neurocognitive functions in long COVID, such as the Mini Mental State Examination (MMSE), MoCA, Rey Auditory Verbal Learning Test, Hopkins Verbal Learning Test, Symbol‐Digit Modalities Test, Brief Visuospatial Memory Test, Boston Naming Test, Trail Making Test, Wisconsin Card Sorting Test, and several batteries from Wechsler Adult Intelligence Scale or Wechsler Memory Scale.^21^, ^30^, ^37^, ^52^, ^53^, ^54^, ^55^, ^56^ For screening, MoCA may be more useful than MMSE to detect potential neurocognition impairment in long COVID cases because of its high sensitivity in detecting even mild cognitive impairment.^54^, ^57^ Regarding mood, anxiety or other subjective symptoms such as fatigue, the PHQ‐9, Beck's Depression Inventory‐II, Beck's Anxiety Inventory, HADS, PTSD Checklist for DSM‐5, General Anxiety Disorder‐7, or Fatigue Severity Scale were used.^25^, ^37^, ^42^, ^52^, ^55^, ^56^, ^58^, ^59^ Thus, the existing scales for cognitive or affective symptoms have mostly been adopted so far, and there is almost no specific battery for long COVID syndrome; however, some psychological stress scales for COVID‐19 pandemic were also developed and used.^60^, ^61^

Since long COVID symptoms occasionally affect multiple areas of neurocognitive functions,^37^ multi‐domain assessment using combined batteries is necessary for accurate evaluation in clinical practice. On the other hand, the objective level of cognitive impairment is generally mild or even insignificant in people with subjective cognitive complaints, while subjective scales such as those for fatigue tend to record quite high scores in long COVID patients.^55^, ^56^ Perhaps, some of the subjective cognitive impairment may be partly attributed to mood or anxiety problems rather than the neurological effect of infection.

### Blood and cerebrospinal fluid examinations

Currently, no specific abnormal findings in the blood or cerebrospinal fluid (CSF) have been established for long COVID, probably because several studies have mainly investigated inflammation‐related markers. Although the extent to which immune system abnormalities persist after COVID‐19 is controversial, it is suggested that abnormalities in the T‐cell system are especially involved and persistent.^62^, ^63^ Clinically, the association of long COVID with interleukin‐6, C‐reactive protein (CRP), neutrophil count, neutrophil‐to‐lymphocyte ratio (NLR), and fibrinogen has been reported.^52^, ^64^, ^65^ Some reports have implicated high NLR as a risk factor for PTSD and low hemoglobin level for depression,^48^ but further validation studies are warranted. As for markers specific to COVID‐19, a low serum level of antibodies against SARS‐CoV‐2 was reportedly associated with severe fatigue and cognitive impairment in patients with long COVID.^66^ Furthermore, in a mouse model, inflammatory cytokines/chemokines were elevated for at least 7 weeks after infection; chemokines with sustained elevations included CCL11, which is associated with neurogenesis and cognitive impairment. Similarly, CCL11 levels were reportedly elevated in human long COVID cases along with the presence of cognitive symptoms.^67^

With respect to CSF findings, although there is a case report wherein SARS‐CoV‐2 RNA was detected in the CSF of a long COVID patient,^68^ another study with a larger cohort did not detect RNA in the patients' CSF samples, even in long COVID cases; hence, long COVID symptoms are not likely to be caused by persistent infection in the central nervous system.^69^ Nevertheless, there is a strong need for biomarker studies to distinguish long COVID syndromes from pre‐COVID condition or concurrent diseases more accurately.^69^

### Neurophysiology

While electroencephalogram (EEG) findings in the acute phase of COVID‐19 showed generalized background slowing at 92.3%, epileptiform discharge at 20.3%, electrographic seizure at 2.05%, status epilepticus at 0.8%,^70^ there are very few neurophysiological studies on long COVID. According to a longitudinal quantitative study on COVID‐19 patients' EEG findings,^34^ increased regional current density and connectivity in the delta band were found to be associated with executive dysfunction at 2 months after hospitalization discharge, and the EEG findings improved at the 10‐month follow‐up. Another study investigated the basis of fatigue symptoms in long COVID, using compound muscle action potential amplitude, resting motor threshold, motor evoked potential (MEP) amplitude, and silent period (SP) duration, which reported abnormal SP shortening and lack of MEP suppression.^71^ Additionally, polyneuropathy or myopathy can be confirmed in some patients, where neurophysiological examinations are useful for diagnosis.^64^

In general, neurophysiological studies on long COVID, especially EEG studies, are lacking, and further investigation is warranted.

### Neuroimaging

While visual abnormalities on brain magnetic resonance imaging (MRI), such as microbleeds, can be identified in some cases,^64^ most cases do not show any specific visually detectable lesions. However, quantitative MRI studies have reported various findings. According to a multimodal imaging study,^72^ cortical thickness can change dynamically but recover to baseline measures at the end of a long‐term follow‐up. Additionally, cortical hypoperfusion was found in severe cases but also tended to recover 10 months after infection, while subcortical nuclei and white matter showed both recoverable and unrecoverable alterations.^72^ Another study reported abnormalities in the white matter hyperintensity volume rather than gray or white matter volumes in COVID‐19 survivors.^34^ As for functional connectivity, a resting‐state functional MRI study revealed abnormal areas including the frontal, parietal and occipital lobes, and thalamus in post‐acute COVID‐19 patients.^73^ Additionally, there are MRI studies on patients with more specific symptoms. Depression and PTSD reportedly correlate with gray matter volumes in the anterior cingulate gyrus or insular cortex as well as axial diffusivity or functional connectivity.^74^ Regarding olfactory dysfunction, improvement in the signal abnormalities and reduced volumes within the olfactory bulbs were observed along with recovery of anosmia.^75^

In addition to MRI studies, brain glucose metabolism measured using 18F‐fluorodeoxyglucose positron emission tomography (PET) may be a biomarker of long COVID syndrome. Some case series studies reported hypometabolism in the cingulate cortex or dysfunction of the locus coeruleus in patients with long COVID and presence of cognitive decline or brain fog.^76^, ^77^ In a large sample study, long COVID patients showed a widespread reduction of metabolism in the bilateral rectal or orbital gyri, right hippocampus and amygdala, brainstem, and cerebellum, which helped to distinguish between patients and healthy controls clearly with 100% accuracy.^78^ This long COVID hypometabolic pattern was replicated using visual assessment in another multicenter study.^79^ Similar hypometabolic patterns in the bilateral medial temporal lobes, brain stem, and cerebellum were also found in pediatric long COVID cases, which suggests similar mechanisms of long COVID syndrome regardless of age.^80^ Another study on long COVID patients detected damages in various other organs, including the lungs, in addition to hypometabolism in the parahippocampal gyrus and thalamus.^81^ Longitudinally, the long COVID metabolic pattern and cognitive dysfunction mostly improved in several months, but some residual abnormality remained.^82^ Given that the brain glucose metabolism can be caused by astroglia as well as neuronal dysfunction, it can be speculated that astroglial inflammation may be involved in brain dysfunction in long COVID cases and could possibly lead to more severe sequelae such as neurodegenerative diseases.^83^ On the other hand, another study reported no significant changes in the brain glucose metabolism in patients with subtle cognitive impairment.^53^

Overall, while neuroimaging studies on long COVID have provided significant and somewhat consistent results, there may be some concerns due to the heterogeneity of patients and self‐reported ambiguous symptoms such as fatigue.^84^

## Potential Mechanism of Long COVID and Its Neuropsychiatric Symptoms

Understanding the mechanisms of long COVID and of cases presenting with neuropsychiatric symptoms would help in the research and development for the prevention and treatment of long COVID. There are many theories regarding the causative mechanisms of long COVID presenting with neuropsychiatric symptoms. In this study, we introduce four potential mechanisms.

SARS‐CoV‐2 enters the human cells by binding to the angiotensin converting enzyme 2 (ACE2) receptor when infecting humans. This receptor is known to be highly expressed not only in the lower respiratory tract but also in parts of the brain, including areas of the somatosensory cortex, rectal/orbital gyrus, temporal lobe, hypothalamus/thalamus, brainstem, and cerebellum.^85^ Direct viral infection of the neuronal cells in these regions occurs by the virus binding to the ACE2 receptor and disrupting the blood–brain barrier.^86^ This invasion causes organic changes in the cells such as demyelination/neurodegeneration and reduction of metabolic activity due to mitochondrial dysregulation. Some researchers have suggested that SARS‐CoV‐2 lies latent in the neurons, resulting in demyelination and neurodegeneration and inducing a greater risk of long‐term effects in some patients recovering from COVID‐19.^87^, ^88^ Although this demyelination hypothesis has still not been proved with strong evidence, these changes have been reported in some cases such as acute disseminated encephalomyelitis following COVID‐19 infection^89^ or combined central and peripheral demyelination following the COVID‐19 vaccination.^90^

Prolonged immune response due to COVID‐19 and the effects of cytokine storms are also possible mechanisms responsible for the neuropsychiatric symptoms of long COVID.^88^, ^91^ Inflammation of the cerebral blood vessels results in blood–brain barrier destruction and immune cell infiltration of the brain cells. In fact, studies reported the signs of reactive astrogliosis in post‐mortem tissue of COVID‐19 patients,^92^ cellular models, and brain organoids.^93^ In fact, brain PET in long COVID patients revealed hypometabolism, which is consistent with astroglial inflammation.^83^ These factors may lead to neuropsychiatric symptoms in long COVID. Moreover, systemic inflammation of the blood vessels throughout the body may cause systemic symptoms of long COVID, which in turn may cause neuropsychiatric symptoms, such as insomnia, depression, and anxiety, as a secondary reaction. Notably, some studies have shown that patients with symptoms of long COVID had higher inflammation level or increased serum cytokine levels.^48^, ^74^, ^94^

Another possibility is that ischemia due to microvascular dysfunction or thrombosis may result in multiple small cerebral infarctions and residual neurological dysfunction.^88^, ^95^, ^96^ In fact, a Finnish study showed that the apolipoprotein E4 (APOE4) carrier status, which is associated with disruption of the blood–brain barrier and is a widely known risk factor for Alzheimer disease,^97^ is an independent risk factor for post‐COVID mental fatigue 6 months after infection.^98^

Additionally, although not a direct cause of long COVID, the pandemic‐related changes and stigma due to COVID‐19 could increase vulnerability to stress. This could result in exacerbation or persistence of psychological symptoms such as depression, anxiety, and insomnia. A previous study showed that patients with psychogenic non‐epileptic seizures had worsened seizures due to increased stress related to the pandemic, even among those who were not directly infected.^99^ An SR has shown that the indirect impact of the COVID‐19 pandemic on the general population's mental health is substantial as is the impact on those with COVID‐19.^100^

## Risk Factors of the Neuropsychiatric Symptoms of Long COVID

Understanding the risk factors that make COVID‐19 patients susceptible to long COVID is an important area of interest for clinicians. A meta‐analysis of the prognostic factors for post‐COVID‐19 syndrome revealed that female sex and severe conditions during acute phase of COVID‐19 are common risk factors for overall post‐COVID‐19 syndrome.^101^ In addition, many other factors and differences in these factors for each neuropsychiatric symptom of long COVID have been reported. Hence, we described each risk factor from the two perspective of patient characteristics and conditions during acute COVID‐19 infection.

One of the major symptoms for long COVID is fatigue as abovementioned. The following patient characteristics were reported as risk factors for fatigue symptoms as long COVID: older age,^98^, ^101^, ^102^, ^103^ APOE ε4 career,^98^ female sex,^104^ chronic pulmonary disease,^105^ and migraine.^106^ Furthermore, the following conditions and interventions during the acute phase of COVID‐19 have been identified as risk factors: more number of symptoms,^104^ constitutional neuropsychiatric symptoms,^104^ hospital admission,^103^ ICU stay,^103^ headache,^107^ neurological complications during hospitalization,^21^ corticosteroid administration during hospital stay,^102^ and intravenous immunoglobulin administration during hospital stay.^102^

Regarding sleep disturbance as a symptom of long COVID, the following risk factors were reported: female sex,^24^ old age,^108^ lower education level,^48^ diabetes,^48^, ^108^ obesity,^109^ and preexisting hypertension.^110^ Additionally, the following conditions and interventions during the acute phase of COVID‐19 were identified as risk factors: severity of COVID‐19,^48^ need for oxygen support,^48^ oxygen saturation on admission,^108^ CRP,^108^ serum ferritin,^108^ and D‐dimer.^108^ In addition, sleep disturbance correlated with depression and anxiety 3 months after discharge.^25^

Cognitive impairment or memory dysfunction is also a common symptom of long COVID. The following patient characteristics were reported as risk factors for cognitive impairment or memory dysfunction: older age,^111^ education ≤12 years,^112^ Black race,^112^ current smoker,^13^ high pre‐hospitalization National Health System score,^113^ and interaction of baseline functional status and unemployment prior to hospitalization.^112^ Moreover, the following conditions during the acute phase of COVID‐19 have been identified as risk factors: non‐admission in ICU,^111^ constitutional neuropsychiatric symptoms and psychological distress,^104^, ^111^ D‐dimer levels,^35^ low arterial oxygen partial pressure/fractional inspired oxygen ratio,^59^ and severe pulmonary disease based on the Brescia‐COVID Respiratory Severity Scale.^113^ On the contrary, a Brazilian cohort study of 425 patients conducted 6–9 months after hospital discharge revealed that psychiatric or cognitive outcomes were neither related to any clinical factors of acute illness severity nor to the psychosocial stressors associated with the illness.^46^

The following factors were reported as risk factors for brain fog as a symptom of long COVID, which is a subjective memory complaint: female sex,^32^ respiratory problems at the onset of COVID‐19,^32^ and ICU admission.^32^

Considering depression or anxiety as a symptom of long COVID, the risk factors frequently differed among studies with contrasting results. The following patient characteristics were associated with an increased risk of depression or anxiety: personality traits,^114^ female sex,^88^, ^101^, ^115^ multimorbidity,^115^ younger age.^115^ In contrast, a cross‐sectional study in Egypt showed that younger age groups, 18 to 30 years old, were at lower risk of depression.^48^ Furthermore, the following conditions during the acute phase of COVID‐19 have been identified as risk factors: hospitalization,^8^ severity of COVID‐19,^116^ diabetes mellitus and low hemoglobin levels,^48^ severity of inflammation,^74^ and brain structure and function.^74^ In contrast, some studies revealed that non‐severity and non‐hospitalization during acute COVID‐19 were associated with increased risk.^8^, ^48^ Another study showed that psychiatric outcomes including depression and anxiety were not associated with acute COVID‐19 severity and disease‐related psychosocial stressors.^46^ Therefore, acute phase of COVID‐19 may not be responsible for depression/anxiety of long COVID.

Regarding PTSD as a symptom of long COVID, the following factors were reported as risk factors: female sex,^88^, ^117^ lower education level,^48^ history of psychiatric disorders,^117^ high NLR in the acute phase,^48^ and anxiety/depression symptoms in the acute phase.^117^ Conversely, in a cross‐sectional survey of 206 patients conducted 4–6 months after discharge in India, none of the patient characteristics and severity of COVID‐19 showed an association with PTSD.^49^

Smell disturbance is a unique symptom of COVID‐19 compared to other virus infection. The reported risk factors for smell disturbance as long COVID, female sex was reported as the risk factor.^13^

The risk factors for headache as a symptom of long COVID, preexisting hypertension^110^ and headache in the acute phase of COVID‐19 were reported as risk factors.^107^

Summarizing these reported risk factors for each symptom, female, age, severity of COVID‐19 in acute phase, and past medical history, were found to be reported as risk factors for some symptoms in many papers.

## Prognosis, Treatment, and Prevention

Most studies have reported that long COVID symptoms, including cognitive dysfunctions, affective disorders, sleep disturbance, olfactory dysfunctions, and headache, could be improved within several months,^21^, ^40^, ^75^, ^118^, ^119^, ^120^ even in pediatric cases.^121^ However, some others reported symptoms persistent for 12 months.^21^, ^122^, ^123^ Particularly, depression/anxiety associated with long COVID may not improve.^21^, ^42^, ^43^ Although not yet understood, the prognosis of long COVID due to brain complication of acute COVID infection such as encephalitis might also be different. The detailed prognosis for each manifestation has been discussed in the ‘Neuropsychiatric manifestations of long COVID’ section. Neuroimaging studies have also found improvement in the brain abnormalities over time in most long COVID cases in terms of cortical thickness changes,^72^ cerebral perfusion,^72^ and glucose metabolism.^82^ Thus, although careful attention is needed for patients with persistent symptoms, it seems that most of the long COVID symptoms alleviate over time.

There have been several attempts or theories to improve the long COVID symptoms, such as rehabilitation for fatigue and cognition,^124^ psychological intervention for anxiety,^125^ indomethacin for headache,^126^ olfactory training or intranasal steroid spray for olfactory dysfunction,^127^, ^128^ cognitive processing therapy for PTSD,^129^ phenytoin for cognitive deficit,^130^ and exercise training for functional and psychological problems.^131^ Most of these treatments reported significant improvement. However, owing to the lack of appropriate control groups, these studies must be considered preliminary. This point is justified by the fact that some percentage of the long COVID symptoms may improve naturally over time. In other words, considering the relatively favorable natural course of long COVID, randomized controlled trials (RCTs) are needed to demonstrate the efficacy and risks of potential therapies. In fact, a previous RCT on corticosteroid nasal spray failed to elicit clear efficacy for post‐COVID anosmia.^132^ However, according to another recent RCT,^133^ adaptogens, a type of herbal medicine, improved the physical symptoms, shortened the duration of fatigue and chronic pain, and reduced serum interleukin‐6 level when compared to the placebo group in long COVID, while it did not show differences in terms of other psychiatric or cognitive performances. Notably, these symptoms also improved, but the same improvement was observed in the placebo group.^133^

Other potential therapeutic targets have also been theoretically proposed, including nuclear factor erythroid‐derived 2‐like 2 and oxidative stress,^134^ amyloid fibrin microclots,^95^ histamine receptors and antagonists,^63^ probiotics,^88^ microvascular dysfunctions,^96^ and cholinergic anti‐inflammatory pathway.^135^ Although these potential targets could be beneficial for patients, invasive treatments are less likely be considered, given the possibility of self‐limited disease course. At the same time, further research should focus on identifying reliable and sensitive biomarkers of risk factors in order to identify potential patients at a risk of severe and/or persistent symptoms and requiring intensive care.

Regarding prevention, COVID‐19 vaccination is effective. Some studies revealed that vaccination reduced the risk of long COVID.^16^, ^136^, ^137^ In particular, compared to one vaccine dose (odds ratio [OR], 0.86; 95% CI, 0.21–3.49, *P* = 0.83), two vaccine doses (OR, 0.25; 95% CI, 0.07–0.87, *P* = 0.03), and three vaccine doses (OR, 0.16; 95% CI, 0.03–0.84, *P* = 0.03) were associated with a lower risk of long COVID.^136^ Hence, as the frequency of vaccine doses increases, the risk of long COVID can decrease. A similar effect has been observed with the omicron variant.^16^ Hence, clinicians should promote COVID‐19 vaccination in order to prevent COVID‐19 as well as long COVID.

## Conclusion

This article comprehensively reviewed the neuropsychiatric aspects of long COVID (Table 1). The neuropsychiatric symptoms of long COVID commonly include fatigue, cognitive impairment, sleep disorders, depression, anxiety, and PTSD. There are no specific tests for long COVID diagnosis, but some characteristic findings, such as hypometabolism on PET, have been reported. The pathogenesis of long COVID can be attributed to inflammation, ischemic effects, direct viral invasion, and social and environmental changes. Some patient characteristics and the severity and complications of acute COVID‐19 may be associated with an increased risk of neuropsychiatric symptoms. Long COVID may resolve spontaneously or persist, depending on the types of neuropsychiatric symptoms. Although there remains a lack of established treatments specifically for long COVID, various psychological and pharmacological treatments have been attempted. COVID‐19 vaccination plays a key role in the prevention of long COVID. With differences among the COVID‐19 variants, including the omicron variant, the symptoms of long COVID are likely to change in the future. Further studies clarifying the aspects of long COVID to develop effective treatments are warranted.

**Table 1:** Summary of the neuropsychiatric aspects of long COVID

Definition	Long COVID includes ongoing symptomatic COVID‐19 and post‐COVID‐19 syndrome Ongoing symptomatic COVID‐19: signs and symptoms of COVID‐19 from 4 to 12 weeks Post‐COVID‐19 syndrome: signs and symptoms that develop during or after an infection consistent with COVID‑19, continue for more than 12 weeks and are not explained by an alternative diagnosis
Neuropsychiatric manifestations (symptoms and prevalence)	Sleep disturbance, 21.4%–34.4%; fatigue, 17.5%–32.9%; objective cognitive impairment, 10.3%–35.7%; anxiety, 13.3%–26.8%; PTSD, 9.9%–24.1%; subjective cognitive impairment, 8.9%–25.0%; depression, 7.5%–21.5%; dysosmia, 8.2%–15.6%; dysgeusia, 4.7%–11.4%; headache, 3.6%–12.0%; sensorimotor disturbances, 2.4%–12.3%; dizziness, 1.6%–5.1%; etc.
Examinations	Psychological assessment tools: general tools (MMSE, MoCA, PHQ‐9, BDI‐II, BAI, GAD‐7, HADS, PCL‐5, TSQ, Fatigue severity scale, etc.) and specific tools (TMDP and others) Blood: changes in neutrophil count, CRP, NLR, IL‐6, or fibrinogen CSF: no specific findings Neuroimaging: various findings on brain MRI and hypometabolism on PET Neurophysiology: increased regional current density and connectivity at delta band on EEG
Risk factors (varies by symptoms)	Patient characteristics: female sex, age, Black race, lower education level, comorbidities, etc. Conditions during acute COVID‐19: severity of COVID‐19, hospitalization, complications, neuropsychiatric symptoms, inflammation, CRP, D‐dimer, types of treatments, etc.
Mechanism	Inflammation, direct invasion, ischemia, social and environmental changes
Variants	Long COVID risk might be different among variants
Prognosis	Symptoms showing improvement: cognitive dysfunctions, affective disorders, sleep disturbance, olfactory dysfunctions, headache, and PTSD Symptoms showing poor improvement: depression and anxiety
Intervention	Psychological intervention, rehabilitation, pharmacological treatment, etc.
Prevention	COVID‐19 vaccination (Frequent vaccinations are more effective)

BAI, Beck Anxiety Inventory; BDI‐II, Beck Depression Inventory‐II; COVID‐19, coronavirus disease 2019; CRP, C‐reactive protein; CSF, cerebrospinal fluid; EEG, electroencephalogram; GAD‐7, General Anxiety Disorder‐7; HADS, Hospital Anxiety and Depression Scale; IL, interleukin; MMSE, Mini‐Mental State Examination; MoCA, Montreal Cognitive Assessment; MRI, magnetic resonance imaging; NLR, neutrophil‐to‐lymphocyte ratio; PCL‐5, PTSD Checklist for Diagnostic and Statistical Manual of Mental Disorders‐5; PET, positron emission tomography; PHQ‐9, Patient Health Questionnaire‐9; PTSD, post‐traumatic stress disorder; TMDP, Tokyo Metropolitan Distress Scale for Pandemic; TSQ, Trauma Screening Questionnaire.

## Author contributions

T.K. contributed to the conception of this article. All authors outlined this article. T.K. conducted a literature searching. All authors investigated previous research. All authors drafted the manuscript and critically reviewed it. Finally, all authors approved this manuscript.

## Disclosure statement

The authors declare no conflict of interest.
